# In silico analysis of atrial fibrillation and hypertension mechanism of action secondary to ibrutinib/acalabrutinib in chronic lymphocytic leukemia

**DOI:** 10.1038/s41598-025-07756-2

**Published:** 2025-07-31

**Authors:** Raúl Córdoba, Antoni Bayés-Genís, Ana Muntañola, Dolors Colomer, Jorge Castro, Carolina Leiva, Esther Álvarez, Eduardo Zatarain-Nicolas

**Affiliations:** 1 Department of Hematology, Fundación Jiménez Díaz University Hospital, Health Research Institute IIS-FJD, Madrid, 28040 Spain; 2 Heart Institute, Germans Trias i Pujol University Hospital, Badalona, CIBERCV Spain; 3 Department of Hematology, Mútua Terrassa, Terrassa University Hospital, Terrassa, Spain; 4 Department of Hematopathology, Clínic Barcelona University Hospital, Institut d’Investigacions Biomèdiques August Pi Sunyer (IDIBAPS), Centro de Investigación Biomédica en Red de Cáncer (CIBERONC), Barcelona, Spain; 5 Medical Department, AstraZeneca Farmacéutica Spain, Madrid, Spain; 6 Cardiology Department, Hospital General Universitario Gregorio Marañón, Instituto de Investigación Sanitaria Gregorio Marañón (IISGM), Centro de Investigación Biomédica en Red Cardiovascular (CIBER-CV), Complutense University, Madrid, Spain

**Keywords:** Bruton’s tyrosine kinase inhibitors, Ibrutinib, Acalabrutinib, Atrial fibrillation, Hypertension, Chronic lymphocytic leukemia., Computational biology and bioinformatics, Cancer, Chronic lymphocytic leukaemia

## Abstract

Ibrutinib and acalabrutinib are first- and next-generation Bruton Tyrosine Kinase inhibitors (BTKi), respectively, approved for chronic lymphocytic leukemia (CLL). Ibrutinib has been associated with cardiovascular events, including atrial fibrillation (AF) and hypertension. Acalabrutinib has demonstrated non-inferior progression-free survival than ibrutinib in relapsed/refractory CLL patients, with a lower cardiovascular event incidence. These adverse events seem to be derived from off-targets rather than BTK inhibition. Machine learning algorithms were applied to identify targets likely to trigger AF and hypertension in simulated CLL patients receiving acalabrutinib or ibrutinib. Common ibrutinib and acalabrutinib off-targets showed association with AF through structural remodeling and electrophysiology/ectopic activity mechanisms (TEC and ERBB4). There was association with hypertension through inflammation (ERBB4) and oxidative stress and endothelial dysfunction (ERBB4 and RIPK2). Ibrutinib-specific off-targets showed association with AF through structural remodeling (HCK, FGR, LYN, FYN, YES1, and FLT3) and electrophysiology activity (LYN and SRC), and with hypertension through inflammation (LCK, JAK3, and FLT3) and oxidative stress and endothelial dysfunction (ERBB2, BLK, SRC, and CSK). No acalabrutinib-specific off-targets were identified for AF or hypertension. This study supports that BTKi off-target selectivity may justify the different AF and hypertension incidences, suggesting their association with several ibrutinib-specific off-targets and identifying no acalabrutinib-specific ones.

**Supplementary Information:**

The online version contains supplementary material available at 10.1038/s41598-025-07756-2.

## Introduction

Bruton tyrosine kinase (BTK) is a non-receptor tyrosine kinase indispensable for B lymphocyte development and differentiation^1–3^which plays a significant role in survival, proliferation, and adhesion of malignant B lymphocytes in chronic lymphocytic leukemia (CLL)^4–7^. BTK inhibitors (BTKi) are currently indicated for the treatment of hematological malignancies^8,9^ and have transformed the management of patients with CLL^10^. However, they can inhibit other tyrosine kinases besides BTK^11,12^causing unfavourable off-target side effects that may even be treatment-limiting.

The first generation BTKi approved for the treatment of CLL was ibrutinib. Despite its survival benefits, there is concern about potentially associated adverse events (AEs) likely derived from its off-target inhibition^13^including hypertension and atrial fibrillation (AF)^14–17^. This concern worsens when considering that CLL usually appears in older adults, with a median age of around 70 years at diagnosis and a high burden of cardiovascular diseases^13,16^. Thus, comorbidities and possible drug-drug interactions need to be considered in clinical practice^18^. The next generation of BTKi, such as acalabrutinib, was designed for greater selectivity in BTK inhibition, leading to fewer off-target effects^19–21^. The phase III ELEVATE RR trial, comparing acalabrutinib with the first-generation ibrutinib in previously treated high-risk CLL patients, demonstrated non-inferior progression-free survival with overall lower risk of cardiovascular AEs^22^.

There is limited data that advances the knowledge of the molecular mechanisms responsible for BTKi therapy-induced cardiovascular events. Systems biology and machine learning have aided in understanding the pathophysiology of several diseases and mechanisms of action of drugs for hematological or cardiovascular diseases, among others^23–25^including the investigations of adverse drug reactions^26,27^. Thus, such an approach could also shed light on the mechanisms of action behind BTKi-induced cardiovascular events and the differences observed between first- and next-generation drugs.

In light of the above, we aimed to identify potential mechanisms behind the BTKi-induced hypertension and AF through an in silico evaluation that used Therapeutic Performance Mapping System (TPMS) technology and focused on the different mechanisms of action between ibrutinib and acalabrutinib.

## Results

### Drug and disease molecular characterization

Our literature search identified the proteins shown in Supplementary Table S3 as direct targets of BTKi. The pathophysiological processes and protein effectors identified are detailed in Supplementary Table S4. From the review of published literature, we also compiled the list of proteins indirectly modulated by acalabrutinib (P_AC_) and proteins indirectly modulated by ibrutinib (P_IB_) (Supplementary Table S5).

**Fig. 1:**
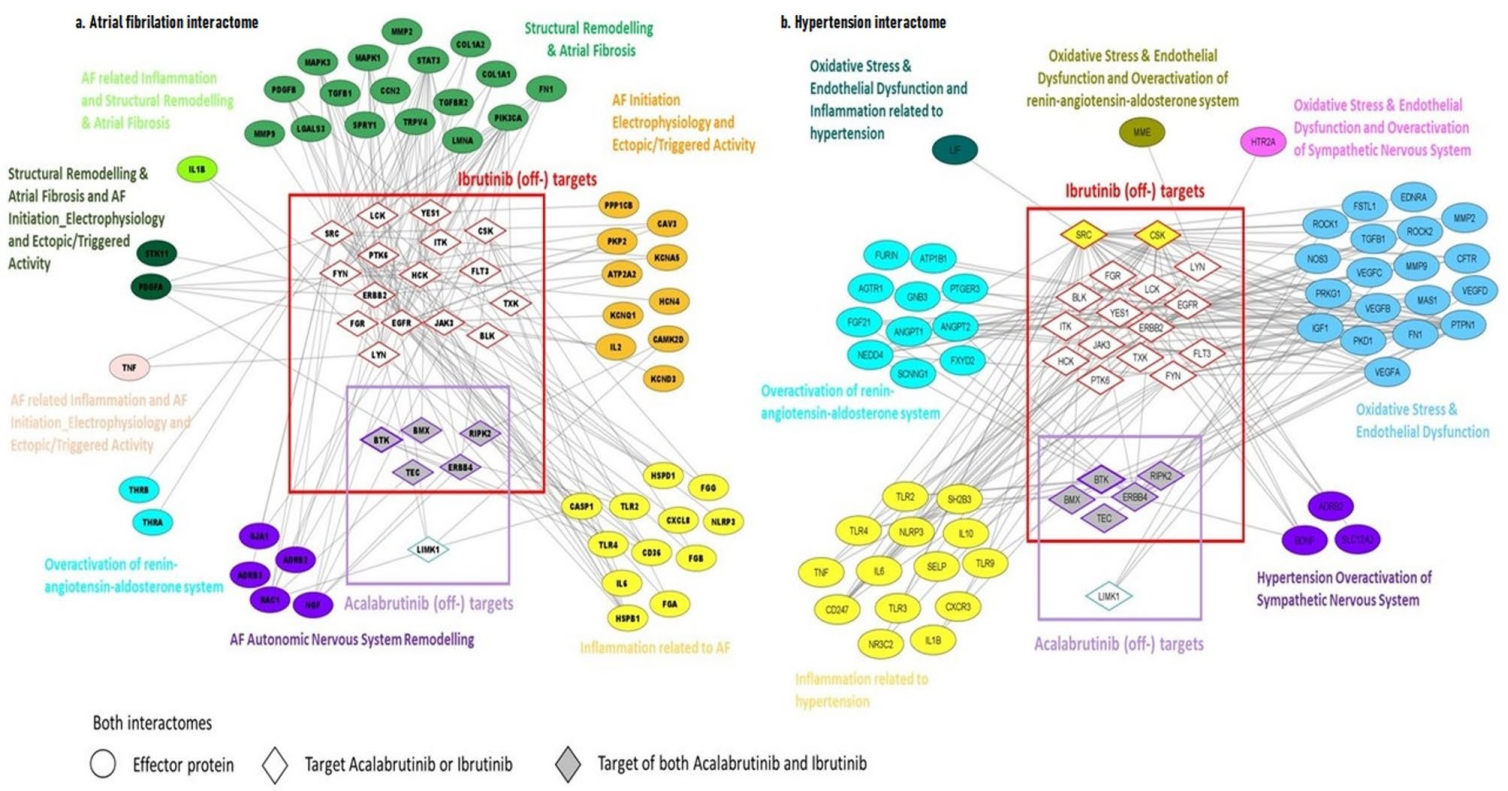
Response network. Human protein networks (interactome) showing the effectors of off-target binding of ibrutinib and acalabrutinib leading to atrial fibrillation (**a**) and hypertension (**b**). Main physiological processes found to be involved in the onset of atrial fibrillation: inflammation; structural remodeling & atrial fibrosis; electrophysiology and ectopic/triggered activity; autonomic nervous system remodeling; overactivation of the renin-angiotensin-aldosterone system; oxidative stress and endothelial dysfunction; and overactivation of sympathetic nervous system. Main physiological processes related to hypertension: inflammation; oxidative stress and endothelial dysfunction; overactivation of sympathetic nervous system; and overactivation of RAAS.

For the characterization of the cardiovascular events, five main pathophysiological processes were found to be involved in the onset of AF induced by ibrutinib and acalabrutinib off-target binding: (1) inflammation; (2) structural remodeling and atrial fibrosis; (3) atrial fibrillation initiation electrophysiology and ectopic/triggering activity; (4) autonomic nervous system remodeling; and (5) overactivation of the renin-angiotensin-aldosterone system (RAAS). Figure 1a provides an overview of the identified effectors and direct protein interactions, grouped by corresponding pathophysiological process. The analysis showed that ibrutinib-specific targets are more connected to several AF pathophysiological processes than acalabrutinib.

Regarding hypertension, our triggering analysis yielded four pathophysiological processes related to hypertension in BTKi-treated patients: (1) inflammation; (2) oxidative stress and endothelial dysfunction; (3) overactivation of sympathetic nervous system; and (4) overactivation of RAAS. Figure 1b provides an overview of the identified effectors and direct protein interactions between them, grouped by corresponding pathophysiological processes.

### BTKi-evoked atrial fibrillation mechanisms modelling and evaluation

The TPMS modelling analysis prioritized common BTKi and ibrutinib-specific targets in triggering AF. Results of the analysis suggested a lack of a role for BTK inhibition in the mechanisms involved in the onset of AF. Numerical values of the analysis are shown in Table 1. Common off-targets TEC and ERBB4 were predicted to induce structural remodeling and atrial fibrosis and electrophysiology and ectopic activity associated with AF. Ibrutinib-specific off-targets HCK, FGR, LYN, FYN, FLT3 and YES1 were predicted to also trigger structural remodeling and atrial fibrosis processes; LYN and SRC were predicted to trigger electrophysiology and ectopic activity; and CSK was predicted to modulate the autonomic nervous system remodeling. No acalabrutinib-specific target was predicted to trigger AF.

**Table 1:** Results obtained from the triggering analysis to evaluate ibrutinib and acalabrutinib target and off-target contribution to atrial fibrillation in CLL. Off-targets that were predicted to contribute to atrial fibrillation are marked in bold.

(Off-) target name	Score (inhibited) – Score (activated)					
	Inflammation	Structural remodeling & atrial fibrosis	Electrophysiology and ectopic/triggered activity	Autonomic nervous system remodeling	Overactivation of the renin-angiotensin-aldosterone system	BTKi
***HCK***	-0.40	**1.27**	0.10	-0.06	-0.31	I
***FGR***	-0.32	**1.05**	0.17	-0.06	-0.29	I
***LYN***	-0.45	**1.02**	**0.30**	-0.06	-0.32	I
***ERBB4***	-0.56	**0.91**	**0.63**	-0.36	-0.55	I/A
***FYN***	-0.20	**0.75**	0.10	-0.80	-0.32	I
***TEC***	-0.10	**0.43**	**0.30**	-0.15	-0.04	I/A
***FLT3***	-0.62	**0.24**	0.18	-0.06	-0.29	I
***YES1***	-0.02	**0.23**	0.04	-0.06	-0.01	I
***CSK***	-1.27	-0.09	-1.30	**0.43**	-0.04	I
***SRC***	-1.48	-2.88	**0.37**	-1.79	-0.50	I
*BMX*	-0.27	-0.17	-0.12	0.04	0.00	I/A
*LIMK1*	0.00	0.00	0.00	0.00	0.00	A
*ITK*	0.00	0.11	0.16	-0.17	0.00	I
*PTK6*	0.11	-0.20	-0.03	-0.01	-0.01	I
*TXK*	0.01	-0.06	0.00	-0.27	0.00	I
*JAK3*	-0.93	-0.39	0.12	-0.16	-0.26	I
*BTK*	-0.41	-0.58	-0.04	-0.30	-0.66	I/A
*LCK*	-1.45	-0.13	0.02	-0.15	-0.28	I
*BLK*	-1.78	-0.60	-0.63	0.05	-0.17	I
*ERBB2*	-1.28	-1.21	-0.23	-0.26	-0.51	I
*EGFR*	-1.16	-2.25	-0.65	-0.16	-0.62	I
*RIPK2*	-1.95	-2.48	-0.92	-0.07	-0.40	I/A

A: acalabrutinib; BTKi: Bruton’s tyrosine kinase inhibitor; CLL: chronic lymphocytic leukemia; I: ibrutinib.

**Fig. 2:**
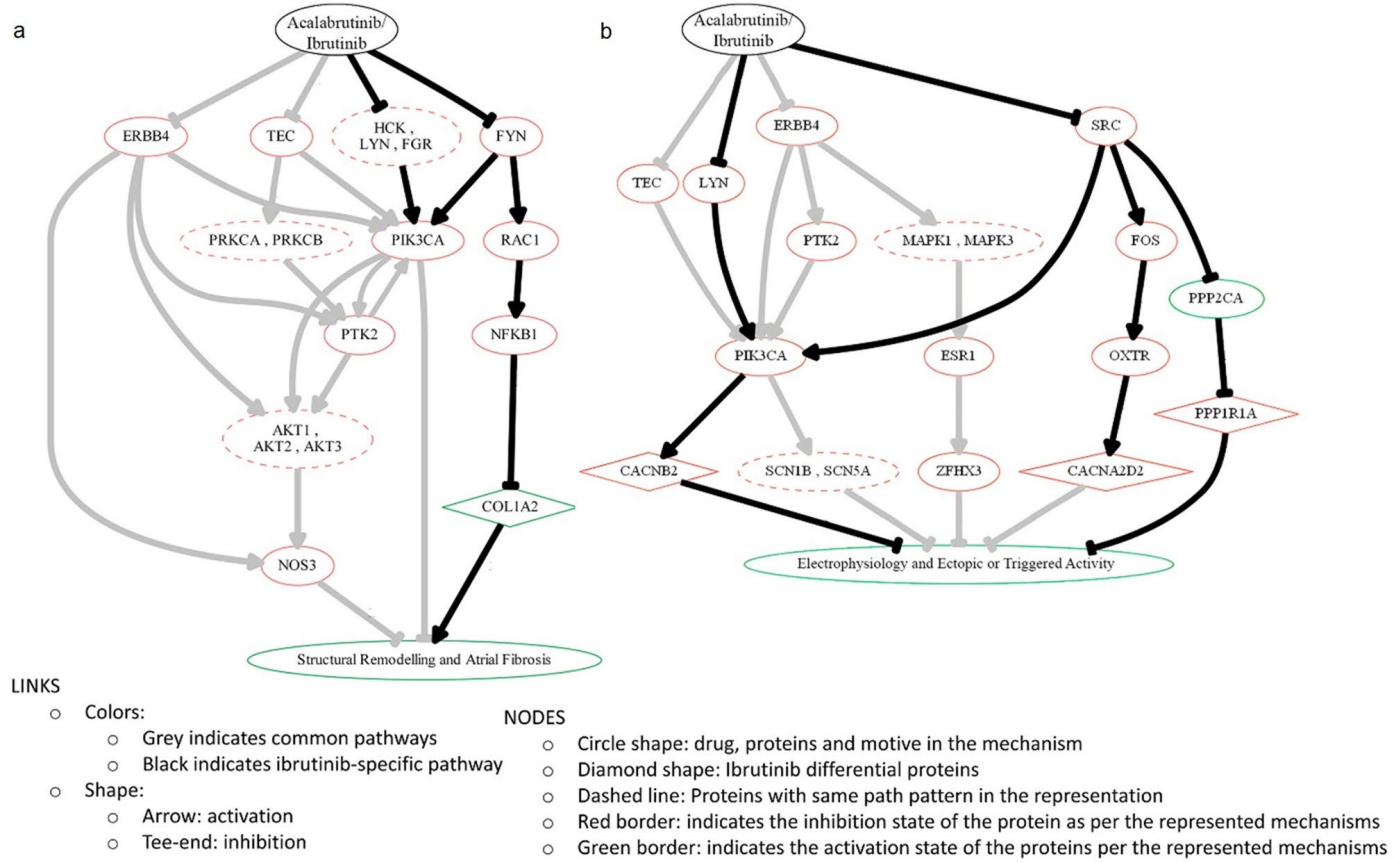
Schematic representation of the predicted differential molecular mechanisms mediating AF secondary to ibrutinib and acalabrutinib. (**a**) shows structural remodeling and atrial fibrosis in AF; (**b**) shows electrophysiology and ectopic or triggered activity in AF. This Figure was created to represent the TPMS mechanism of action prediction by using Graphviz software (https://graphviz.gitlab.io/). See Supplementary Fig. S1 for references supporting the represented interactions.

Further modelling of the main and differential pathways by which ibrutinib and acalabrutinib may trigger AF was performed using TPMS technology for each of the pathophysiological processes and drugs. In this additional analysis, we considered the detected off-targets contributing to AF as a stimulus, while the other targets were considered as inhibited. Figure 2 depict the schematic representation of the predicted differential molecular mechanisms mediating ibrutinib- and acalabrutinib-induced AF (see Supplementary Fig S1 and Supplementary Table S7 for information related to each represented link). According to the models built, both drugs were able to modulate common pathways through their common off-target binding. However, ibrutinib more strongly modulated proteins involved in AF, such as COL1A2, CACNB2, CACNA2D2 and PPP1R1A (Fig. 2a and b). Among the proteins for which a BTKi effect has been described according to the literature (P_AC_ and P_IB_) and that are present with predicted protein activity in at least one of the AF models, 94% of the P_IB_ and 100% of the P_AC_ were predicted to be modulated in the AF models in the same direction (activated or inhibited) as described in the literature (Supplementary Table S5).

We evaluated whether cotreatments used in CLL patients could interfere with the predicted BTKi-evoked AF mechanisms considering the impact of co-administration over the tSignal according to the AF molecular definition (Supplementary Table S6). In general, the difference in tSignal induced by the cotreatments was low (i.e., close to 0) and shared a common tendency among both BTKis evaluated. Also, most of the evaluated drugs presented a differential effect < 0, i.e., the cotreatments were not predicted to contribute to increase the signal of the BTKi over AF. The cotreatments more likely to interfere with the AF mechanisms were identified considering statistical significance and a minimum tSignal difference when co-administered with the BTKi (|Diff Effect|>0.05). These drugs were antiarrhythmics (namely, amiodarone, betablockers and Ca^2+^ channel blockers), ADP receptor inhibitors, corticoids, and angiotensin receptor blockers. All these cotreatments were predicted to potentially reduce the BTKi-evoked AF mechanisms. Although the tendency was similar for both BTKi, a stronger effect seemed to be detected in acalabrutinib models, especially for betablockers and angiotensin receptor blockers.

### BTKi-evoked hypertension mechanisms modelling and evaluation

TPMS modeling analysis suggested a lack of role for BTK inhibition in triggering hypertension. Numerical values of the analysis are shown in Table 2. Related to hypertension, common off-targets RIPK2 and ERBB4 were predicted to induce oxidative stress and endothelial dysfunction, and the latter also induced inflammation (Table 2). Ibrutinib-specific off-targets LCK, JAK3, and FLT3 were predicted to trigger inflammation processes related to hypertension; ERBB2, BLK, SRC, and CSK were predicted to trigger oxidative stress and endothelial dysfunction; and CSK were predicted to trigger the RAAS overactivation. No acalabrutinib-specific target was predicted to trigger hypertension.

**Table 2:** Results obtained from the triggering analysis to evaluate ibrutinib and acalabrutinib target and off-target contribution to hypertension in CLL. Off-targets that most contribute to hypertension are marked in bold.

(Off-) target name	Score (inhibited) – Score (activated)				
	Inflammation	Oxidative stress and endothelial dysfunction	Overactivation of sympathetic nervous system	Overactivation of the renin-angiotensin-aldosterone system	BTKi
***ERBB4***	**0.24**	**0.60**	-0.29	-0.30	I/A
***ERBB2***	0.01	**1.39**	-0.18	-1.30	I
***RIPK2***	-1.62	**1.30**	-0.21	0.18	I/A
***BLK***	-1.40	**0.80**	-0.27	-0.11	I
***SRC***	-0.79	**0.43**	-0.46	-0.35	I
***LCK***	**0.27**	-0.36	-0.15	-0.52	I
***JAK3***	**0.40**	-0.63	-0.24	-0.51	I
***FLT3***	**0.26**	-0.79	-0.12	-0.60	I
***CSK***	-1.15	**0.25**	-0.04	**1.14**	I
*LIMK1*	0.00	0.00	0.00	0.00	A
*TXK*	0.00	-0.06	0.00	0.02	I
*PTK6*	0.06	-0.09	-0.01	-0.19	I
*YES1*	-0.21	-0.12	-0.02	-0.13	I
*ITK*	-0.01	-0.19	0.04	-0.09	I
*LYN*	-0.01	-0.43	-0.15	-0.65	I
*EGFR*	-0.56	-0.46	-0.20	-1.72	I
*BTK*	-1.36	-0.47	0.00	-0.48	I/A
*TEC*	-0.01	-0.49	-0.04	-0.46	I/A
*BMX*	-0.37	-0.58	0.03	0.03	I/A
*FYN*	-1.04	-0.60	0.11	-0.24	I
*FGR*	-0.02	-0.67	-0.12	-0.45	I
*HCK*	-0.23	-0.71	-0.12	-0.47	I

A: acalabrutinib; BTKi: Bruton’s tyrosine kinase inhibitor; CLL: chronic lymphocytic leukemia; I: ibrutinib.

**Fig. 3:**
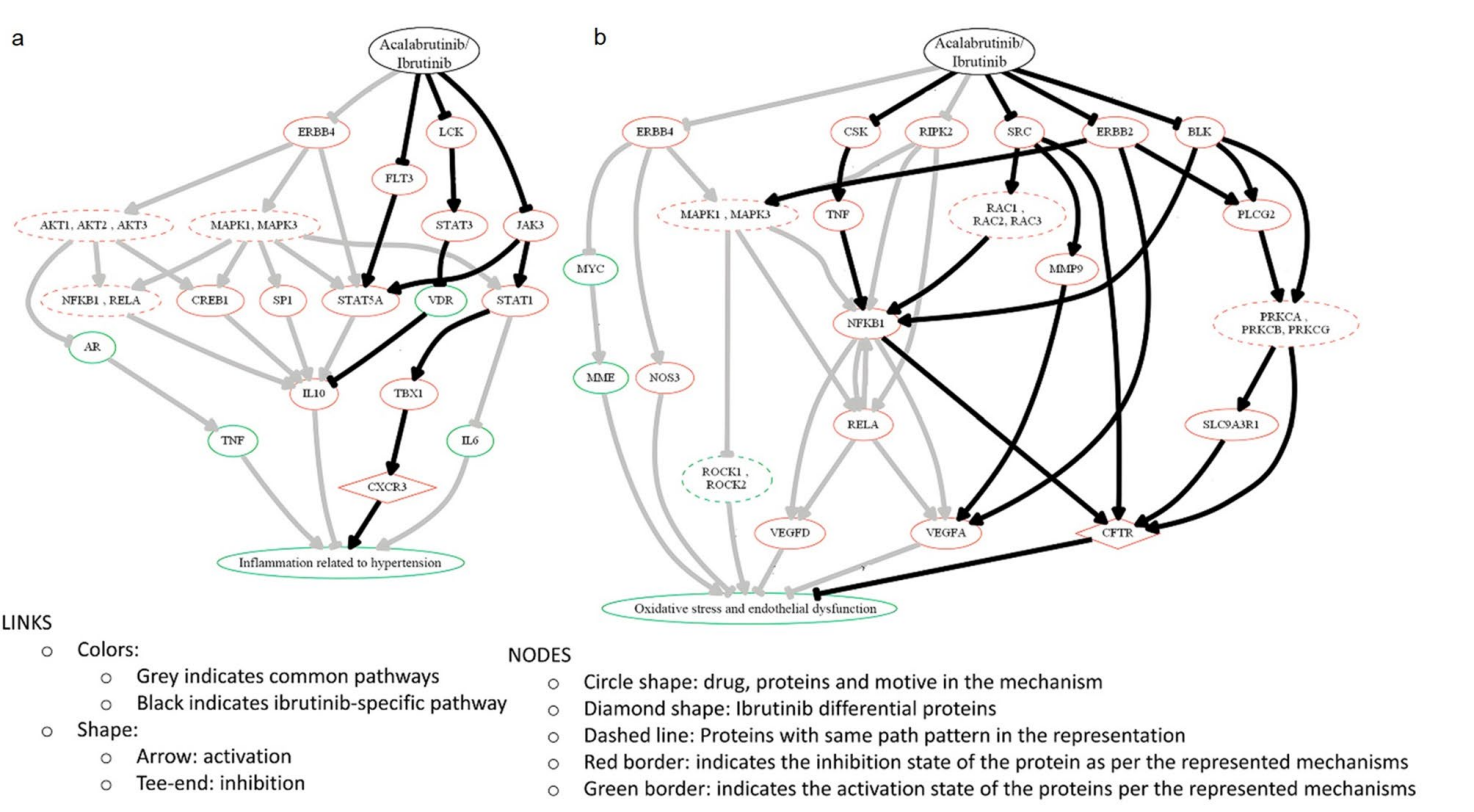
Schematic representation of the predicted differential molecular mechanisms mediating hypertension secondary to ibrutinib and acalabrutinib. (**a**) shows inflammation related to hypertension; (**b**) shows oxidative stress and endothelial dysfunction in hypertension. This Figure was created to represent the TPMS mechanism of action prediction by using Graphviz software (https://graphviz.gitlab.io/). See Supplementary Fig. S2 for references supporting the represented interactions.

Similarly to AF, further TPMS modelling of the main differential pathways by which ibrutinib and acalabrutinib may lead to hypertension was performed. Figure 3a and b depict the schematic representation of those predicted differential molecular mechanisms mediating hypertension through inflammation and oxidative stress, respectively (see Supplementary Fig. S2 and Supplementary Table S7 for information related to each represented link). According to the models built, both drugs were able to modulate common pathways through their common off-target binding, but ibrutinib modulated more strongly proteins involved in hypertension, such as CXCR3 and CFTR (Fig. 3a and b). Regarding hypertension models, 94% of the P_IB_ and 71% of the P_AC_ were predicted to be modulated in the same direction (activated or inhibited) as described in the literature (Supplementary Table S5).

Our co-treatment analysis results showed that virtually no cardiovascular disease treatments were predicted to have worsening effects upon BTKi-induced hypertension mechanisms (Supplementary Table S6). As from the AF analysis, most of the evaluated drugs presented a Diff effect < 0, i.e., the cotreatments were not predicted to contribute to increase the signal of the BTKi over hypertension. When checking the potential of other drugs to affect BTKi-induced hypertension mechanisms, only one drug class, the Ca^2+^ channel blockers, showed a tSignal above the threshold set, particularly potentially reducing the ibrutinib-induced mechanism over the oxidative stress and endothelial dysfunction. None of the evaluated treatments showed an effect on the inflammation for hypertension. Importantly, none of the evaluated cotreatments were detected to increase the signal association to BTK inhibition over hypertension, according to the mechanistic models created.

## Discussion

Our in silico analysis showed that BTK inhibition seems to be unrelated to the molecular triggers of AF or hypertension. They may partly result from common off-target effects mediated mainly by TEC and ERBB4 for AF or RIPK2 and ERRB4 for hypertension. Our models propose ibrutinib-specific mechanisms mediated by ibrutinib-specific off-targets involved in AF (structural remodeling and atrial fibrosis mediated by HCK, FGR, LYN, FYN, YES1 and FLT3; and electrophysiology regulation mediated by LYN and SRC) and in hypertension (inflammation mediated by LCK, JAK3 and FLT3; and oxidative stress and endothelial dysfunction mediated by ERBB2, BLK, SRC and CSK). However, no acalabrutinib-specific mechanism was detected, which aligns with the previous studies that report a lower incidence of cardiovascular events in CLL patients receiving acalabrutinib^8,9,22^.

Even though they share common mechanisms, the selectivity of acalabrutinib with less identified off-targets may explain its better safety profile. For instance, ibrutinib might potentiate the blockade of phosphoinositide 3-kinase (PIK3) pathway through its exclusive off-targets HCK, FGR, FYN and LYN, which have been reported to activate PIK3^28^. In support of our hypothesis, ibrutinib has been shown to inhibit the PI3K/Akt pathway, including indirect downregulation of PI3K-p110a^28–30^and reduced PIK3CA activation has been related to increased susceptibility to AF and cardiac conduction deterioration^31,32^. Inhibition of PI3K-p110a has also been linked to consequent vascular tissue fibrosis and cellular remodeling (factors well established to underlie hypertension development) in histologic cardiovascular tissue from subjects with arrhythmogenic cardiotoxicity^29,32^. ERBB4/HER4 is expressed in the heart and its downregulation has a major impact on cardiomyocyte function^33^. A comparative analysis showed that ibrutinib inhibits several kinases implicated in causing cardiovascular side effects, such as HER2, HER4, and TEC, while acalabrutinib inhibits HER4, but not HER2, and only slightly TEC^12,32^. For ibrutinib, the binding to HER2 has been previously implicated as a candidate for cardiovascular adverse events^22,34^. Furthermore, both drugs, through the inhibition of TEC and ERBB4, might reduce nitric oxide production, promoting atrial endothelial dysfunction, enhanced cardiac hypertrophy and fibrosis, and reduced capillary formation^35^. Ibrutinib and acalabrutinib, through their inhibitory effects over TEC and ERBB4, might promote the downregulation of the calcium channel CACNB2, favoring electrical remodeling^36^. According to these results, ibrutinib can differentially downregulate CACNB2 through its inhibitory effect over its exclusive off-targets LYN and SRC, which might block the subsequent activation of PIK3CA, resulting in a more profound downregulation of CACNB2^31^. In accordance with the findings by Xiao et al.^37^. our models suggest CSK as an ibrutinib off-target with potential to trigger cardiovascular events, particularly through autonomic nervous system remodeling, oxidative stress, and RAAS modulation. However, given the regulatory nature of CSK over several tyrosine kinases signaling cascades^38^our approach did not allow to detect CSK inhibition-mediated mechanisms, as the ibrutinib effect on other off-targets acting downstream of CSK seems to block CSK-mediated mechanisms of RAAS overactivation and results in a neutral effect. The AF and hypertension induction mechanisms detected in our analysis suggest rapid (inflammation, electrophysiology changes) and long-term changes (fibrosis), which is in accordance with ibrutinib-induced changes observed in rodent models^37^as well as endothelial dysfunction observed in vitro^39^.

CLL is a disease of aged and comorbid patients in whom polypharmacy is common and so the probability of drug-to-drug interaction. This may deter the patient’s response to the drug or increase adverse events^40^. Our models were built to assess whether the most frequently used comorbidity therapies impacted on the ibrutinib and acalabrutinib mechanisms involved in the development of AF and hypertension. The results showed that some antiarrhythmics, antiplatelets, RAAS inhibitors and anti-inflammatory drugs (most of them used as anti-hypertensive drugs in clinical practice) could interfere and potentially reduce AF occurrence as could be expected. However, no such effect was clearly observed for hypertension, except for some mild effects on oxidative stress and endothelial dysfunction of calcium channel blockers. This diverse impact can be attributed to the specific pathophysiological mechanisms governing each condition. While, in the case of BTKi-evoked AF, inflammation, electrophysiology and structural remodelling play pivotal roles, BTKi-evoked hypertension involves other mechanisms such as persistent endothelial dysfunction, increased vasoconstrictor activity, and heightened sympathetic nervous system activity. The mild effect of calcium channel blockers on hypertension is likely to primarily addresses endothelial dysfunction through vasodilation, while their antiarrhythmic effect would have a more relevant activity on AF.

According to our model, calcium channel blockers and corticoids might diminish the signal of both ibrutinib and acalabrutinib mechanisms associated with AF. These beneficial effects might be mainly attributed to the alleviation of inflammatory processes that accompany AF^41,42^. Non-dihydropyridine (non-DHP) calcium channel blockers have demonstrated efficacy in controlling cardiac rhythm in patients with AF^43^although our results must be interpreted with caution because the approach we applied considers drugs as a “protein target profile” as defined in databases and literature (Supplementary Table S1). In the case of calcium channel blockers, although two types can be found according to their chemical nature (dihydropyridines – DHP – and non-DHP), the proteins described to be targeted by both categories are the same; thus, the approach applied cannot differ between them, but the antiarrhythmic effect showed in our study would be presumed for non-DHP calcium channel blockers.

However, the primary route for metabolism and elimination of ibrutinib is through cytochrome P450 3 A (CYP3A)-mediated metabolism, which also plays a prominent role in the metabolism of numerous other medications^44^including the above-mentioned non-DHP calcium channel blockers. In this sense, clinical guidelines advise that co-administration of ibrutinib^8,40^ or acalabrutinib^9,40^ with strong CYP3A inhibitors or inducers should be avoided. Ultimately, healthcare professionals should be aware of the probability of drug-drug interactions when administering BTKis. Overall, our analysis suggests that the other co-administered drug therapies might not directly affect the BTKi-induced mechanisms to produce cardiovascular events.

Besides the limitations commented on throughout the discussion, our approach, as all in silico modelling strategies^23,26^is limited by the information available about drugs and diseases, and consequently some assumptions had to be made, as some drug-induced mechanisms might be not so well established in the literature. Therefore, the mechanisms shown in this article outline the generated models, prioritizing those more frequent in the universe of solutions, though the effects of some off-targets could have been overlooked due to lesser general knowledge of their functions. Thus, it cannot be discarded a joint role of other off-targets on the described mechanisms and specific pathways impact within the described general mechanisms, such as the myocardial ion channels within the electrophysiology and ectopic approached in this project. Also, the differential molecular mechanisms found for ibrutinib are limited by the criteria used to identify them and additional drugs not considered in the analysis might also interact with the studied drugs, and the cardiovascular processes assessed. Despite these limitations, our generated models present accuracies against the training set about 93% with respect to the current published knowledge, supporting the accuracy and confidence of our approach. Although BTKi molecular target profile characterization was based on a structured literature search, no systematic review was conducted. In addition, the different times from drug discovery and use, which might affect the amount of available information (i.e., more published work for ibrutinib than for acalabrutinib), might lead to literature bias downplaying the target profile associated to acalabrutinib. To prevent this bias, we did not include information on molecular changes associated to each drug (P_IB_ and P_AC_) on the model building process. Nevertheless, we found that our models were able to reproduce between 71% and 100% of the information compiled.

In conclusion, this model supports that BTKi-induced AF or hypertension are rather derived from off-target effects partly mediated by TEC and ERBB4 for AF, and RIPK2 and ERRB4 for hypertension. As no acalabrutinib-specific off-targets were identified to trigger AF or hypertension, the identified ibrutinib-specific off-targets involved in these events could explain its association with their previously reported higher incidence rates and explain potential mechanisms that make patients under ibrutinib treatment more prone to AF and hypertension. Despite the limitations associated to a computational modelling strategy, our findings are a starting point to understand the lower incidence of AF and hypertension associated with acalabrutinib compared to ibrutinib in CLL patients. Further research on these cardiovascular events considering the clinical validation of the identified mechanism of action, the additive effect of known clinical risk factors, other relevant co-treatments that might not have been considered in the analyses, and all currently available BTKi warrant further assessment in the clinical setting to predict individual patient risk and develop predictive models in BTKi-treated populations.

## Methods

### Study design

**Fig. 4:**
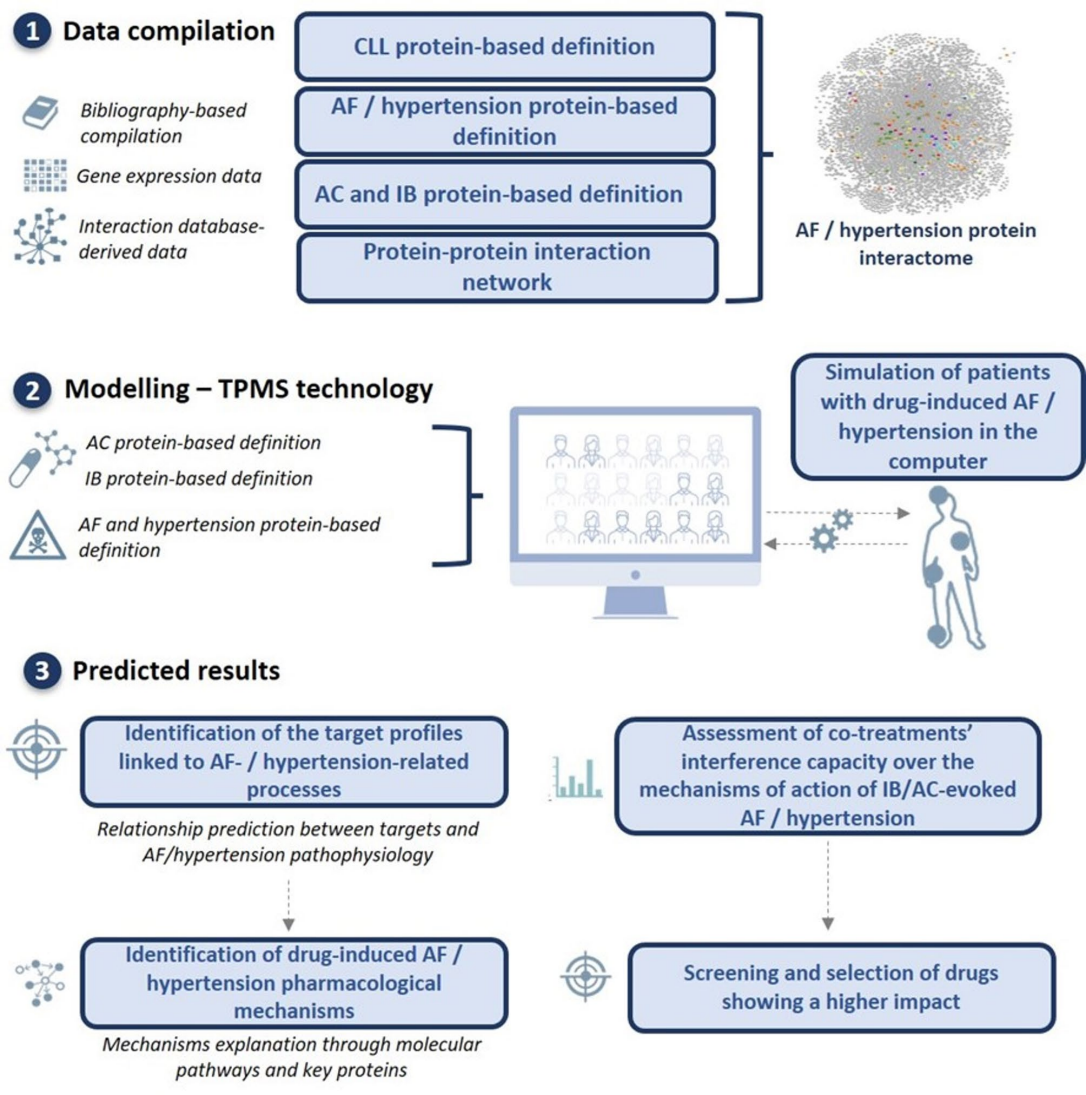
Overview of the in silico TMPS-based analysis methodology used to unravel the mechanisms underlying atrial fibrillation (AF) and hypertension during ibrutinib and acalabrutinib treatment. The main phases employed to simulate the mechanisms of action of Bruton Tyrosine Kinase inhibitors (BTKi) included: (1) data compilation for molecular characterization of BTKi-induced AF and hypertension and modelling of the protein interactome for the two adverse events; (2) mathematical model generation using the validated top-down systems biology- and artificial intelligence-based TPMS approach; and (3) identification of target profiles linked to AF- and hypertension-related pharmacological mechanisms and interference analysis of cotreatments. AC: acalabrutinib; AF: atrial fibrillation; CLL: chronic lymphocytic leukemia; IB: ibrutinib.

In the current study, we applied a top-down systems biology approach based on machine learning upon the human protein-protein interaction network to build and analyze drug mechanisms of action. To do so, we performed the following three steps: (1) Drug and disease molecular data compilation: database and literature-based review to characterize CLL, the cardiovascular events of interest (i.e., hypertension and AF), and the BTKi drugs (i.e., ibrutinib and acalabrutinib) under study; (2) Drug-disease mechanisms modelling: integration of the compiled information with the human protein-protein interaction network applying TPMS (Anaxomics Biotech S.L., Barcelona, Spain) technology^26^ to obtain systems biology-based drug mechanism of action models; and (3) Analysis of the predicted results: evaluation of the potential role of BTKi targets in the cardiovascular events occurrence, obtention of model-derived measures and statistical analyses to identify significant model results to propose mechanistic hypotheses and explain differences among the studied drugs. Additionally, the models were evaluated to assess whether other commonly used cotreatments could affect the predicted mechanisms of action (Fig. 4).

### Disease and cardiovascular adverse events characterization

We intended to determine the targets of BTKi involved in cardiovascular events, as well as explore the mechanisms behind them and evaluate the impact of cotreatment interferences. With this aim, we molecularly characterized CLL, AF, and hypertension following the previously reported methodology^45^.

Briefly, we first performed a structured search in the PubMed database for relevant publications on the molecular pathogenesis, pathophysiology, and molecular mechanisms of the conditions (see Supplementary Methods for more details). Second, we retrieved the publications identified in the searches and assessed them at the title and abstract level. If molecular information describing the condition pathophysiology was present, we thoroughly reviewed the full text and expanded the publication by reviewing relevant references. The aim was to identify the main pathophysiological processes known to be involved in the conditions. Third, each pathophysiological process was further characterized at the protein level. We also reviewed the retrieved publications to identify protein/gene candidates to be condition effectors: proteins whose activity (or lack thereof) was functionally associated with the development of AF or hypertension. At this step, additional PubMed searches were performed specifically on potential protein effector candidates, including all protein names according to UniProtKB (Universal Protein Resource Knowledgebase) database codes, to obtain functionally robust evidence. Candidates were identified as effectors following the previously mentioned criteria.

### Drug characterization

To characterize the BTKi target profile, we reviewed the specialized databases DrugBank^46^ and Stitch^47^along with ibrutinib and acalabrutinib regulatory agencies documentation (European Medicines Agency)^8,9^. We also reviewed scientific literature in PubMed such as original articles and reviews (see Supplementary Methods for more details). Off-targets were selected if reported in the drug’s European Public Assessment Report or in the scientific articles with an IC50 < 500 nM. From the published literature review, we also compiled a list of proteins whose activity or expression is affected by the BTKi, due to drug downstream effect but not being a direct drug target effect. These proteins are hereinafter called P_AC_ and P_IB_.

To elucidate possible drug-to-drug interactions, we reviewed the most frequent comorbidities in CLL patients. We compiled a list of commonly used drugs in these patients, including those for the treatment of cardiovascular diseases, according to clinical practice. Drugs sharing a common mechanism of action were molecularly characterized as drug classes, defined by their main class target, according to DrugBank database information. Supplementary Table S1 shows the list of possible comorbid disease treatments, and the molecular definition used according to DrugBank.

### Systems biology-based modelling by TPMS technology

TPMS technology, based on systems biology and machine learning, was used to evaluate ibrutinib and acalabrutinib targets/off-targets, and to explore the potential mechanisms linking these targets to the AF and hypertension molecular definitions. TPMS integrates available protein-protein interaction network information along with physiological and pathophysiological data to create models similar to a Multiplayer Perceptron of an Artificial Neural Network over the human protein network (where neurons are the proteins, and the edges of the network are used to transfer the information). Models were trained using a compendium of biological and clinical data defining human physiology, as previously described^26,27,45^. This procedure generates a universe of plausible solutions, which conforms a sampling methods-based model. The set of solutions is explored to get the most probable solutions according to the distribution. Supplementary Table S2 summarizes the data used for model construction and training set.

Triggering analysis was performed to explore the role of ibrutinib’s and acalabrutinib’s targets and off-targets as individual proteins in triggering a specific pathophysiological process involved in the studied cardiovascular events. The signal was propagated from each of the proteins under evaluation (i.e., BTKi targets) through the sampling methods-based models. The number of proteins of the processes evaluated that are stimulated with the sign assigned in the characterization at distances 1, 2 and 3 was measured and protein proximity was then positively pondered. Distance here is considered as the number of interactions in the protein-protein network (if two given proteins are directly related, distance is considered as 1; if two given proteins are related through a third protein, distance is considered as 2; and so on). A mean value was obtained for the universe of solutions. The results are presented normalized by the maximum value obtained and expressed as a triggering score. The triggering score of both inhibiting and activating the targets was calculated and the subtraction of the two factors was used to prioritize the targets whose inhibition, rather than overall modulation, led to the pathophysiological process. The target/off-targets were considered to potentially induce the pathophysiological process when [Score (inhibited)] – [Score (activated)] > 0.2.

Using the targets identified through the triggering analysis, and considering the pathophysiological processes triggered as a response, we created BTKi cardiotoxicity mechanism of action models. When a stimulus and a response are set, a mechanism of action model can be created as previously described^26^. The models were built so the rest of ibrutinib and acalabrutinib targets were kept in an inhibited state, to better reproduce the drug effects. The sampling methods-based models allow exploration of the predicted protein activity for each protein within the models (ranging between [-1,1]) and identify the most frequent pathways occurring between a stimulus and the biological process definition.

For cotreatment interference evaluation, when a biological process (i.e., disease or pathophysiological processes involved in the studied cardiovascular events) is defined as a protein set, the direct impact of a stimulus over it can be assessed through the tSignal (ranging between [-1,1])^26^. tSignal is defined as the average predicted protein activity arriving at the protein effectors involved in the protein set, corrected by the sign expected for the protein in the set (Eq. 1). The mentioned stimulus assessed can be the one used for building the TPMS model (i.e., the drug’s targets), or another stimulus not included in the model. Thus, we can evaluate the cotreatments over the created model for the identified targets.

1 $${\rm tSignal} = \:-\frac{1}{n}\sum\:_{i=1}^{n}{v}_{i}{y}_{i}$$

Where n is the number of proteins defining the protein set; $\:{v}_{i}$ are the protein signs (active/inactive) according to each disease/comorbidity definitions; and $\:{y}_{i}$ are the resulting modelled signal values achieved by each protein “i” after stimulating the model with the corresponding stimulus.

The quality (predictive and descriptive capacity) of the models was checked against the training set, with model accuracy defined as the percentage of compliance with this information^26^. Additionally, the predicted protein activity state (activation or inhibition) of the P_AC_ and P_IB_ in the models for each cardiovascular event were checked, and the percentage of agreement with literature information was calculated to assess whether the predicted protein activity of known BTKi effects was correctly modelled. Although TPMS models were protein-based, the interactome where they were built included gene and RNA regulation data. Thus, for standardization purposes, we used gene names to refer to all genes/proteins mentioned in this manuscript when referring to model results.

### Statistical analysis and computational resources

BTKi mechanisms sampling methods-based models were compared between ibrutinib and acalabrutinib to identify differentially modulated effectors, using Wilcoxon rank-sum test (non-parametric) and Benjamini-Hochberg false discovery rate (FDR) for multi-test corrections as described in Supplementary Methods, and applying a previously described classification accuracy^26^.

The impact of treatments most commonly co-administered to CLL patients for their comorbidities over BTKi mechanism models was also evaluated through tSignal comparison^24^as described in Supplementary Methods.

All simulations and analyses were executed using Matlab function and Python or R package (Supplementary Methods). Cytoscape version 3.3.1^48^ and Graphviz (https://graphviz.gitlab.io) were used for visualization purposes.

## Electronic supplementary material

Below is the link to the electronic supplementary material.

Supplementary Material 1

## Data Availability

Data from this article can be found either in the main text figures/tables or in the supplementary material provided. For further inquiries contact the corresponding author.
